# Effect of Dexmedetomidine in Preventing Postoperative Side Effects for Laparoscopic Surgery: A Meta-Analysis of Randomized Controlled Trials and Trial Sequential Analysis (PRISMA)

**DOI:** 10.1097/MD.0000000000002927

**Published:** 2016-03-11

**Authors:** Guoqi Wang, Licheng Zhang, Shenghan Lou, Yuxiang Chen, Yanxiang Cao, Ruirui Wang, Lihai Zhang, Peifu Tang

**Affiliations:** From the Department of Orthopedics (GW, LCZ, SL, YUC, YAC, LHZ, PT), Chinese PLA General Hospital; and Beijing BOE Display Technology Co. Ltd. (RW), BDA, Beijing, P.R. China.

## Abstract

Dexmedetomidine (DEX) has been used extensively for patients during surgery. Some studies found that DEX could reduce the incidence of postoperative side effects in laparoscopic surgical patients. However, no firm conclusions were made about it.

The authors searched for randomized controlled trials (RCTs) in PubMed, EMBASE, and the Cochrane Central Register of Controlled Trials testing DEX administrated in laparoscopic surgical patients and reporting on postoperative nausea, vomiting, shivering, heart rate, mean arterial pressure (MAP), or extubation time after surgery or within 1 hour in postoperative care unit. Trial sequential analysis (TSA) was used for RCTs comparing DEX with placebo or no treatment in laparoscopic surgery patients. A protocol for this meta-analysis has been registered on PROSPERO (http://www.crd.york.ac.uk/prospero) and the registration number is CRD42015020226.

Fifteen studies (899 patients) were included. DEX could significantly reduce the incidence of postoperative nausea (risk ratio [RR] and 95% confidence interval [CI], 0.43 [0.28, 0.66], *P* < 0.0001), vomiting (RR and 95% CI, 0.36 [0.18, 0.72], *P* = 0.004), shivering (RR and 95% CI, 0.19 [0.11, 0.35], *P* < 0.00001), rescue antiemetic (RR and 95% CI, 0.18 [0.07, 0.47], *P* = 0.0006), and increase the incidence of dry mouth (RR and 95% CI, 7.40 [2.07, 26.48], *P* = 0.002) comparing with the control group. In addition, firm conclusions can be made on the results of postoperative nausea according to the TSA. Meta-analysis showed that DEX group had a significantly lower heart rate (mean difference [MD] and 95% CI, −14.21 [−18.85, −9.57], *P* < 0.00001) and MAP (MD and 95% CI, −12.35 [−15.28, −9.42], *P* < 0.00001) than the control group, and firm conclusions can be made according to the TSA. No significance was observed on extubation time between 2 groups (MD and 95% CI, 0.70 [−0.89, 2.28], *P* = 0.39).

The results from this meta-analysis indicated that perioperative DEX decreased postoperative nausea and shivering in laparoscopic surgical patients. However, common adverse effects were lower heart rate and MAP. Firm conclusions cannot be made on postoperative shivering, rescue antiemetic, and dry mouth until more RCTs were included.

## Abstract 2

Supplemental Digital Content is available in the text

## INTRODUCTION

Nausea, vomiting, and shivering are common discomfort after anesthesia and operation, which may cause serious complications without any proper disposal.^1,2^ It is reported that the incidence rate of postoperative nausea and vomiting (PONV) is 70% to 80% in patients of high-risk groups^3^ and shivering is 40% to 60% in regional anesthetic patients.^4^

Many drugs used for the treatment of PONV include butyrophenones, benzamides, histamine-receptor antagonists, and so on. Dexmedetomidine (DEX) is a new α2 agonist. As an effective adjuvant to multimodal analgesia, DEX has been extensively used for patients during surgery.^5,6^ The main role includes sedation, anesthetic-sparing, analgesia, and sympatholytic properties.^7^ Recently, some new studies have pointed that perioperative DEX administration could reduce the incidence of PONV and shivering.^8,9^ In 2012, Blaudszun et al^6^ performed a meta-analysis about effect of perioperative α2 agonists on postoperative morphine consumption and pain intensity. However, patients undergoing various surgeries were included, and only a few studies provided nausea, vomiting, and shivering information. In 2014, a meta-analysis made by Liu et al^10^ indicated that DEX may not be appropriate solely for the purpose of the prevention of postoperative shivering due to the high price and potential adverse events, but they also included patients undergoing various surgeries and did not make a subgroup analysis on different surgeries. It was reported that laparoscopy was one of the main factors associated with an increased risk of postoperative PONV.^11^ Obviously, it is more appropriate to limit patients undergoing a specific kind of surgeries when studying the benefits of DEX.

It remains unclear that whether perioperative DEX administration could reduce the incidence of nausea, vomiting, shivering, or other side effects in all kinds of surgeries or in a specific surgical procedure. In addition, α2 agonist-related adverse effects, such as bradycardia and arterial hypotension, may throw doctors into fear to use it frequently. Our meta-analysis includes 15 randomized controlled trials (RCTs), and identifies the precise effect of DEX on the primary outcomes of PONV, shivering, heart rate, and mean arterial pressure (MAP) for patients undergoing laparoscopic surgeries only. In addition, trial sequential analysis (TSA) was used for RCTs comparing DEX with placebo or no treatment in laparoscopic surgery patients.

## METHODS

### Protocol and Registration

This meta-analysis of RCTs was performed according to the PRISMA (Preferred Reporting Items for Systematic Reviews and Meta-Analyses) recommendations.^12^ This study was not a human or animal experiment, thus ethical approval was not necessary. A protocol for this meta-analysis has been registered on PROSPERO (http://www.crd.york.ac.uk/prospero) and the registration number is CRD42015020226.

### Inclusion and Exclusion Criteria

This meta-analysis would include studies if they met the following criteria: patients >18 years of age who had undergone laparoscopic surgery; DEX versus placebo or no treatment; primary outcomes including postoperative nausea, vomiting, shivering, heart rate, MAP, or extubation time; the outcomes were reported in both DEX group and placebo or no treatment group; and was a RCT. The exclusion criteria were as follows: abstracts, letters, or meeting proceedings; unavailability of full text.

### Search Strategy

Two authors (GW and LCZ) searched PubMed, EMBASE, and the Cochrane Central Register of Controlled Trials according to the guidelines in the Cochrane Handbook.^13^ The search strategy was: “Dexmedetomidine AND Laparoscopic AND (RCT or randomized controlled trial)” or “(Dexmedetomidine or α2 agonist) AND Laparoscopic AND (RCT or randomized controlled trial)” without any restriction to year or language of publication. In addition, we manually searched the reference lists of the included studies for potentially eligible studies. The last search was performed on May 30, 2015, and all results were imported into Endnote X7 (Thomson Reuters, New York, NY). Titles, abstracts, and full texts of potentially relevant articles were screened after excluding duplicated ones. A third reviewer (SL) would be invited if there were any disagreements.

### Data Extraction

All available and relevant data from the included studies were extracted independently by 2 authors. The extracted general data included author, year, and country of publication; sample size; and type of surgery in DEX and control groups. Main outcomes included events of postoperative nausea, vomiting, shivering, rescue antiemetic, dry mouth, heart rate, MAP, and extubation time after surgery or within 1 hour in postoperative care unit (PACU) in DEX and control groups. The extubation time was defined as the time between discontinuation of inhalation agents and extubation. Outcomes after the highest DEX dose administration were extracted when a trial studied different DEX doses.

### Risk of Bias and Methodological Quality Assessment

Two authors assessed all RCTs according to the Cochrane risk of bias tool,^13^ which included 7 categories: random sequence generation; allocation concealment; blinding of participants and personnel; blinding of outcome assessment; incomplete outcome data; selective outcome reporting; and other sources of bias. Each category included 3 levels: low risk, unclear risk, and high risk. In addition, Grading of Recommendations Assessment, Development, and Evaluation (GRADE) guideline was used to evaluate the quality of evidence for postoperative nausea, vomiting, shivering, rescue antiemetic, dry mouth, heart rate, MAP, and extubation time for risk of bias, inconsistency, indirectness, imprecision, and publication bias.^14,15^ They were classified as very low, low, moderate, or high quality.

### Statistical Analysis

Outcomes were estimated by calculating the pooled risk ratio (RR) (95% confidence intervals [CIs]) for dichotomous ones and mean differences (MD) (95% CIs) for continuous ones by RevMan software (version 5.1; Cochrane Collaboration, Copenhagen, Denmark). A *P* < 0.05 was considered statistically significant. Heterogeneity was assessed by visual inspection of the forest plot combined with the results of the test for heterogeneity and the I^2^ test.^16^ Fixed-effects model were used for outcomes with low heterogeneity (I^2^ ≤ 40%). Otherwise, the random-effects model of DerSimonian and Laird^17^ would be selected. Sensitivity analyses were performed by STATA 12.0 (StatCorp, College Station, TX) when heterogeneity was observed in main outcomes. Those data from ≥2 trials would be included in analysis of an outcome.

Type I errors may appear in meta-analyses owing to an increased risk of random error, although a few data were analyzed.^18^ TSA was used to this meta-analysis in order to assess the risk of type I errors (program version 0.9 beta). When the cumulative Z curve in results exceeds the TSA boundary, a sufficient level of evidence for the anticipated intervention effect may have been reached and no further trials are needed. However, if the Z curve does not exceed the TSA boundaries and the required information size has not been reached, evidence to get a conclusion is insufficient. We used one-sided tests, type I error set at 5%, and power set at 80%. The required information size was calculated based on a relative risk reduction (RRR) of 20% in main outcomes.

## RESULTS

### Included Studies and Characteristics

**FIGURE 1:**
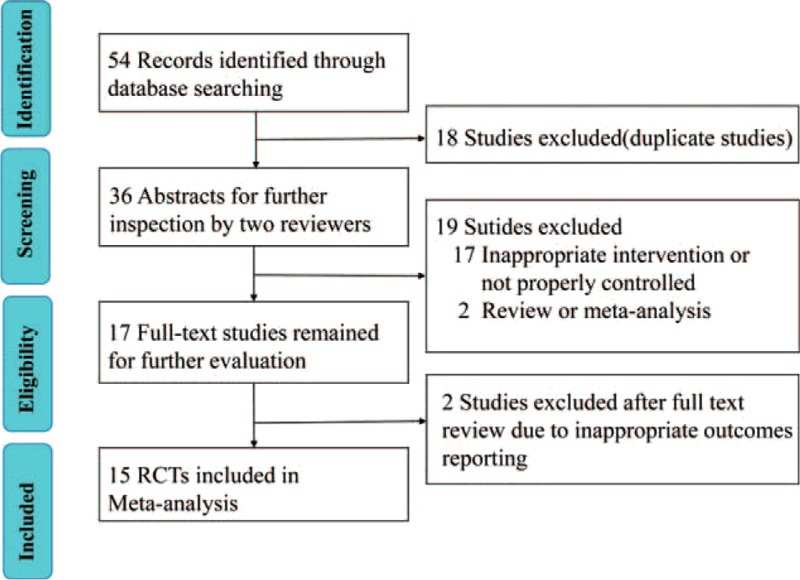
Flow diagram shows the process of literature selection.

The literature selection process is shown in Figure 1. Fifty-four potential articles were obtained initially. Eighteen articles were excluded because they were duplicates. Another 19 articles were excluded due to inappropriate intervention, not properly controlled or article type. Two studies were excluded after full-text review because of inappropriate outcomes reporting. Finally, Fifteen RCTs^8,9,19–31^ published between 1992 and 2015 met the inclusion criteria and were included in this meta-analysis.

Fourteen RCTs compared DEX with placebo (saline). One RCT compared DEX with no treatment.^8^ Eight RCTs had >2 groups.^8,9,19,21,27–29,31^ A total of 899 patients undergone laparoscopic surgeries were included in the meta-analysis. A total of 451 patients were administrated with DEX and 448 were with placebo or no treatment. The sample size in each study ranged from 45 to 120. More details were shown in Table 1.

**TABLE 1:**
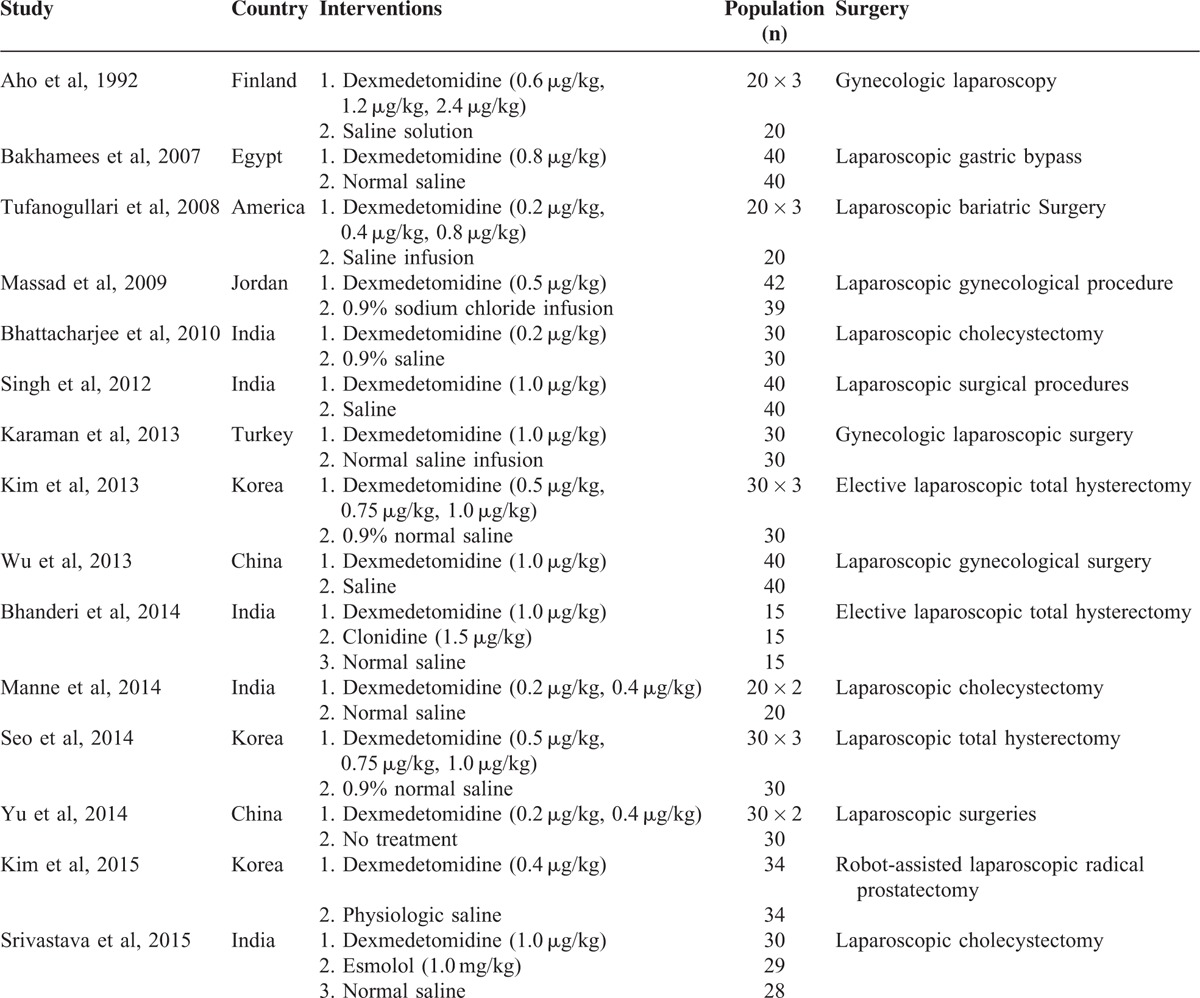
Characteristics of Included Randomized Controlled Trials

### Risk of Bias and Methodological Quality

**FIGURE 2:**
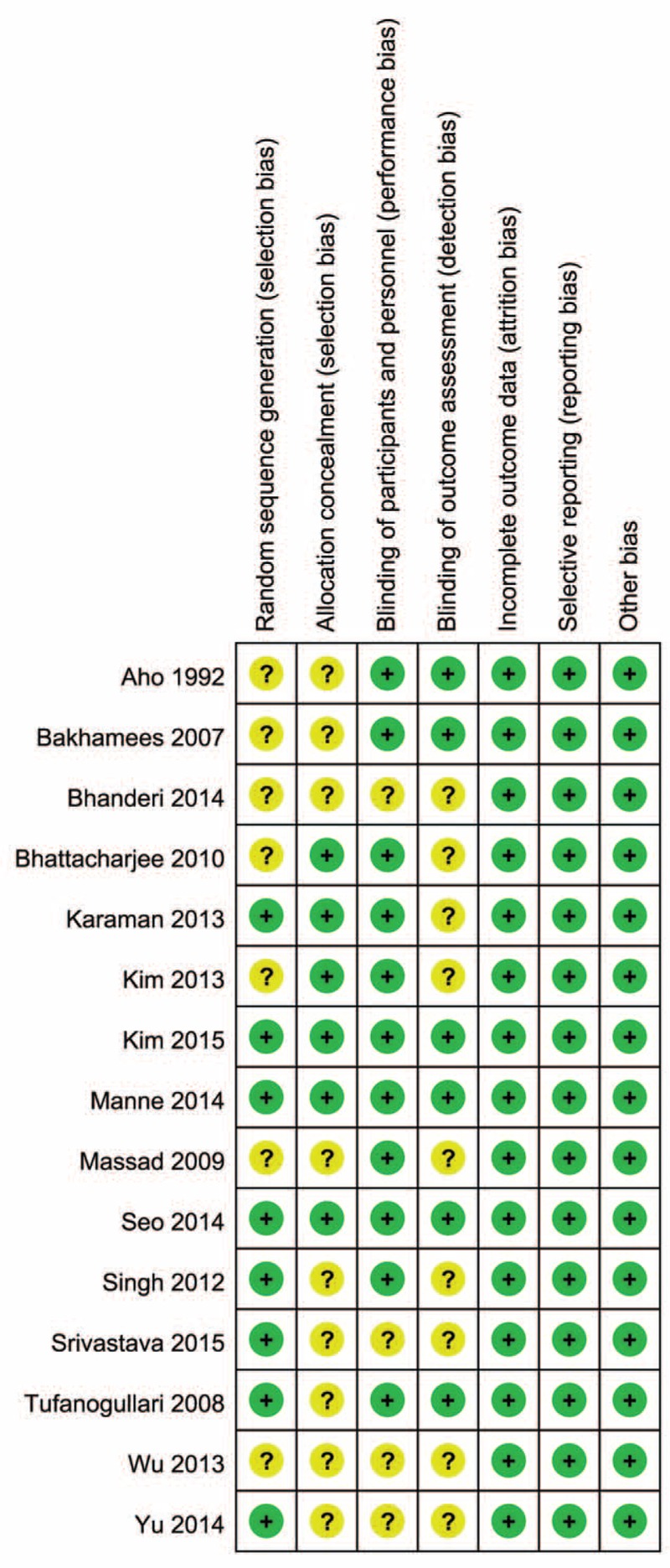
The methodological quality of the RCTs.

Eight trials^8,21,24,25,28–31^ had random sequence generation by computer-generated random number table, computer-coded envelopes, or block randomization technique with opaque sealed envelopes. Six trials had concealment of allocation.^9,23,25,28–30^ Blinding of participants and personnel was present in 11 trials,^9,19–25,28–30^ 6 of which accomplished blinding of outcome assessors.^19–21,28–30^ No incomplete outcome data, selective reporting, or other sources of bias were found in the included studies. On the whole, 3 of all the included trials represented high-quality trials (Figure 2). The GRADE analysis indicated that the quality of the main outcomes was moderate (Table 2). The most common reasons for the decreased level of evidence were suspected publication bias because of inadequate included original studies. Heterogeneity was the reason that reduced the evidence grade of the heart rate, MAP, and extubation time results.

**TABLE 2:**
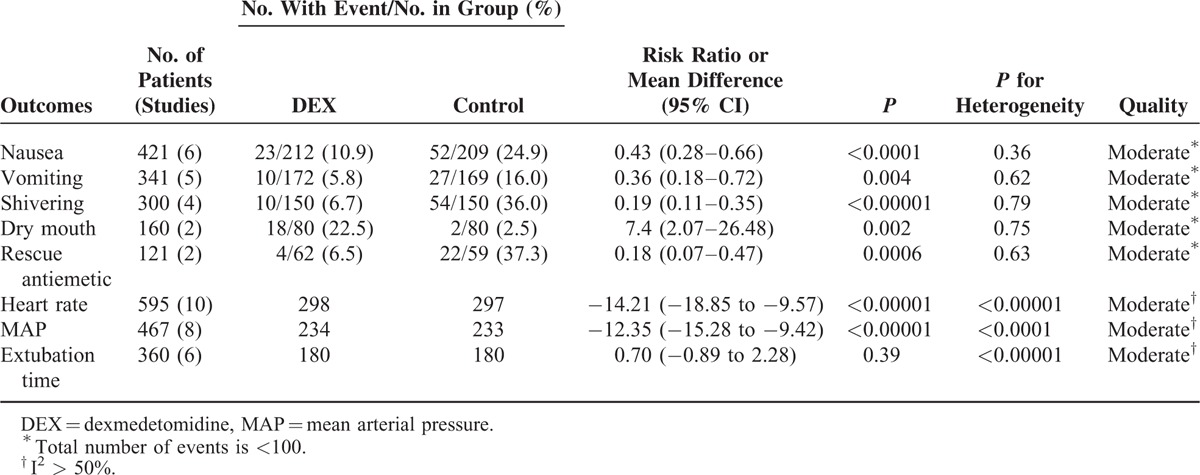
The GRADE Evidence Quality for Outcomes

### Clinical Outcomes

#### Postoperative Nausea, Vomiting, Shivering, Rescue Antiemetic, and Dry Mouth

**FIGURE 3:**
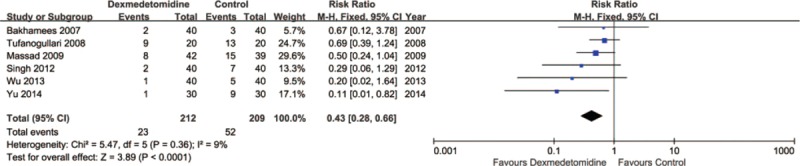
Nausea.

**FIGURE 4:**
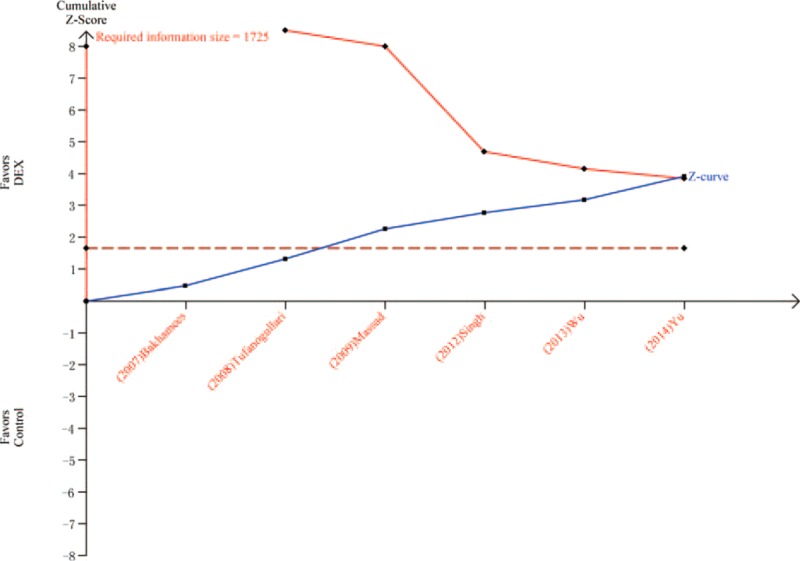
Trial sequential analysis of nausea.

Six trials^8,20–22,24,26^ including 421 patients investigated the antinausea efficacy of DEX compared with control. The incidence of postoperative nausea in the DEX group was significantly lower than in the control group (10.9% vs 24.9%, respectively; RR = 0.43; 95% CI: 0.28–0.66, *P* < 0.0001, I^2^ = 9%) (Figure 3). The TSA indicated that Z curve crossed both the conventional boundary and the TSA boundary (Figure 4).

**FIGURE 5:**
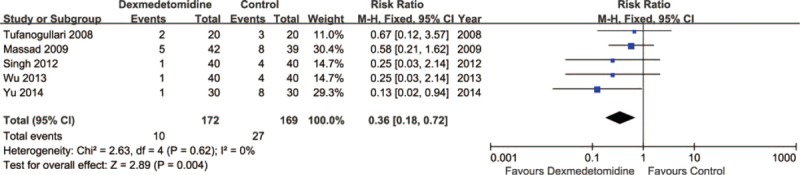
Vomiting.

**FIGURE 6:**
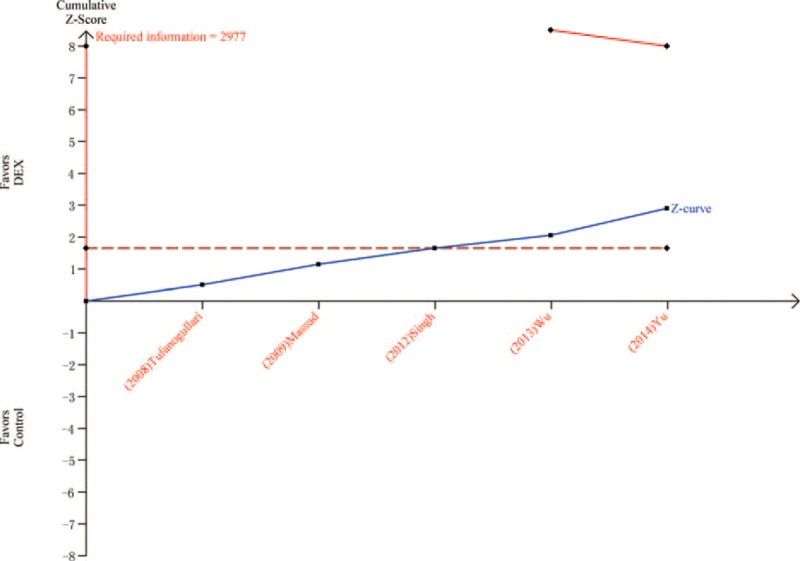
Trial sequential analysis of vomiting.

Five trials^8,21,22,24,26^ including 341 patients investigated the antivomiting efficacy of DEX compared with control. The incidence of postoperative vomiting in the DEX group was significantly lower than in the control group (5.8% vs 16.0%, respectively; RR = 0.36; 95% CI: 0.18–0.72, *P* = 0.004, I^2^ = 0%) (Figure 5). The TSA indicated that Z curve crossed the conventional boundary and did not cross the TSA boundary (Figure 6).

**FIGURE 7:**
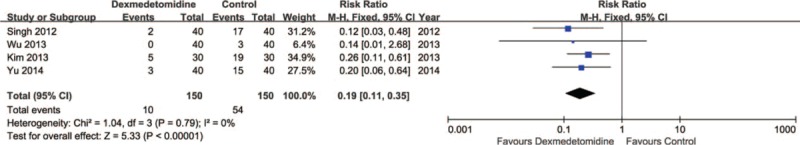
Shivering.

**FIGURE 8:**
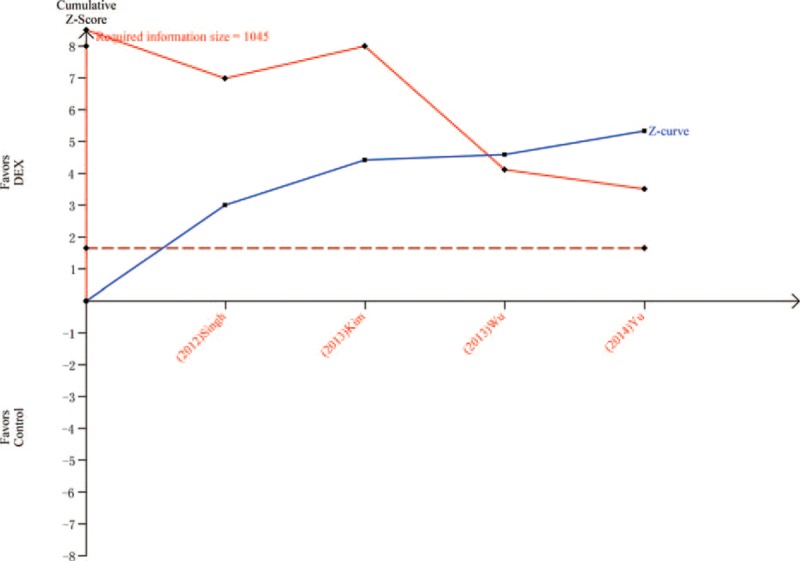
Trial sequential analysis of shivering.

Four trials^8,9,24,26^ including 300 patients investigated the antishivering efficacy of DEX compared with control. In addition, the incidence of postoperative shivering in the DEX group was significantly lower than in the control group (6.7% vs 36.0%, respectively; RR: 0.19; 95% CI: 0.11–0.35, *P* < 0.00001, I^2^ = 0%) (Figure 7). The TSA indicated that Z curve crossed both the conventional boundary and the TSA boundary (Figure 8).

Furthermore, DEX can significantly reduce postoperative rescue antiemetic compared with control (6.5% vs 37.3%, respectively; RR of 2 trials^21,22^: 0.18; 95% CI: 0.07–0.47, *P* = 0.0006, I^2^ = 0%) (Table 2). The TSA indicated that Z curve crossed the conventional boundary and did not cross the TSA boundary (Electronic Supplementary Material, ESM 1). DEX, however, increased the incidence of dry mouth compared with the control (22.5% vs 2.5%, respectively; RR of 2 trials^24,26^: 7.40; 95% CI: 2.07–26.48, *P* = 0.002, I^2^ = 0%) (Table 2). The TSA indicated that Z curve crossed the conventional boundary only (ESM 2).

#### Postoperative Heart Rate, MAP, and Extubation Time

**FIGURE 9:**
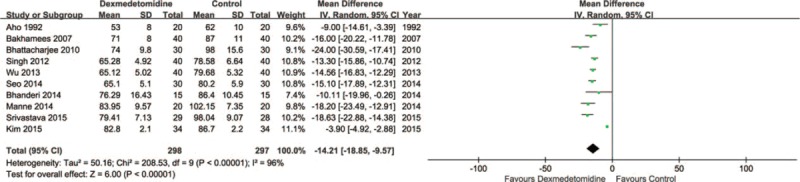
Heart rate.

**FIGURE 10:**
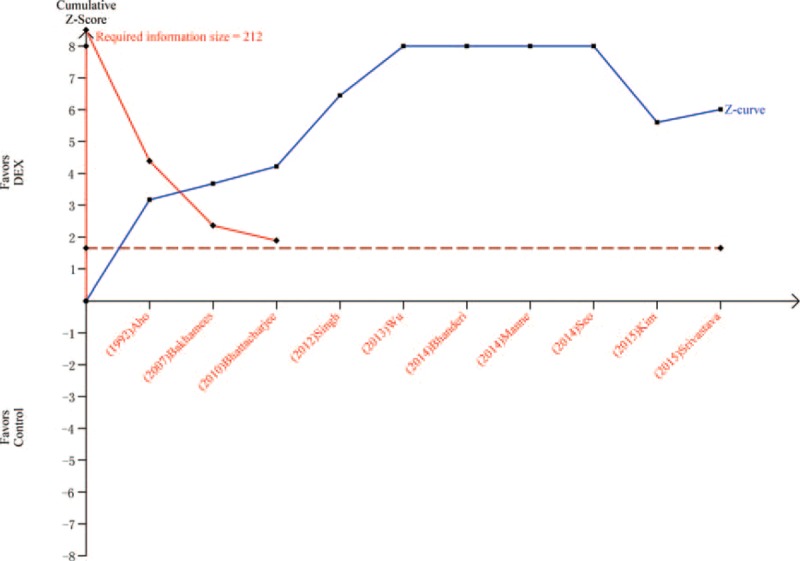
Trial sequential analysis of heart rate.

Ten trials^19,20,23,24,26–31^ including 595 patients compared heart rate in patients treated with DEX or control after surgery or within 1 hour in PACU. Meta-analysis showed that a significantly lower heart rate was associated with DEX (MD = −14.21, 95% CI: −18.85 to −9.57, *P* < 0.00001, I^2^ = 96%) (Figure 9). The TSA indicated that Z curve crossed both the conventional boundary and the TSA boundary (Figure 10).

**FIGURE 11:**
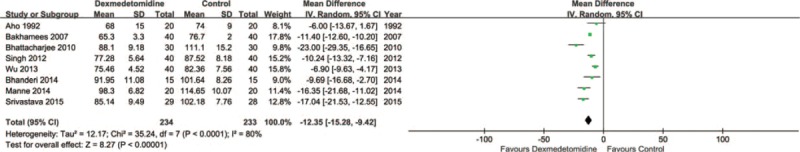
Mean arterial pressure.

**FIGURE 12:**
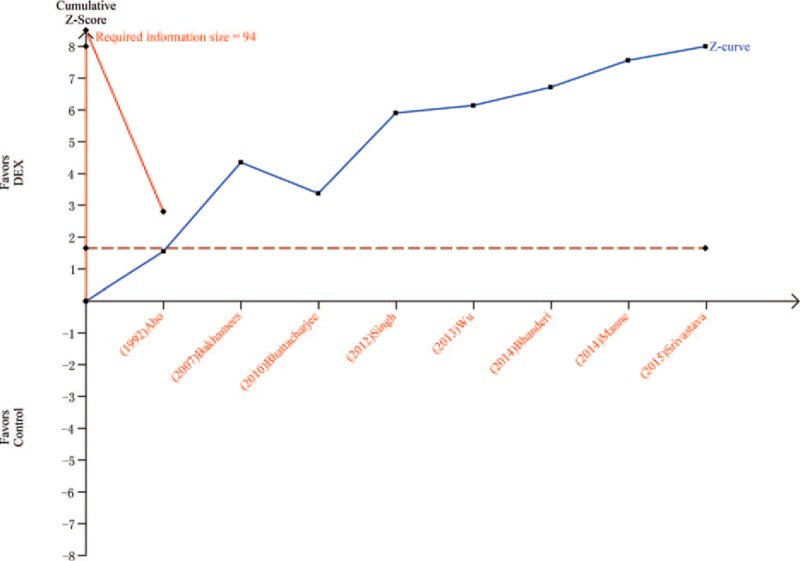
Trial sequential analysis of mean arterial pressure.

Eight trials^19,20,23,24,26–28,31^ including 467 patients compared MAP in patients treated with DEX or control after surgery or within 1 hour in PACU. The MAP was 81.2 mm Hg in DEX group and 93.8 mm Hg in control group. Meta-analysis showed that a significantly lower MAP was associated with DEX (MD = −12.35, 95% CI: −15.28 to −9.42, *P* < 0.00001, I^2^ = 80%) (Figure 11). The TSA indicated that Z curve crossed both the conventional boundary and the TSA boundary (Figure 12).

Six trials^9,20,21,23,25,29^ including 360 patients compared extubation time in patients treated with DEX or control. No significance was observed between 2 groups (MD = 0.70, 95% CI: −0.89 to −2.28, *P* = 0.39, I^2^ = 98%) (Table 2). The TSA indicated that Z curve crossed the conventional boundary only (ESM 3).

#### Publication Bias

Publication bias was not identified for postoperative nausea, vomiting, shivering, rescue antiemetic, and dry mouth. Publication bias was observed in heart rate, MAP, and extubation time (ESM 4, ESM 5, ESM 6).

### Sensitivity Analyses

Heterogeneity was observed in heart rate, MAP, and extubation time (I^2^ > 40). Sensitivity analyses showed that omitting any single study did not significantly affect the results of heart rate and MAP (ESM 7, ESM 8). However, DEX group had a longer extubation time than control group after omitting “Bakhamees 2007” (ESM 9).

## DISCUSSION

This meta-analysis was aimed to evaluate the impact of perioperative systemic usage of DEX on postoperative nausea, vomiting, shivering, rescue antiemetic, dry mouth, heart rate, MAP, and extubation time. The control group was placebo or no treatment.

Comparing with the control group, DEX could reduce the prevalence of postoperative nausea from 24.9% (control group) to 10.9%. This result is similar with some previous studies.^6,10^ Bakri et al^32^ indicated that DEX could reduce the incidence and severity of postoperative nausea. However, TSA in this article showed that firm conclusion can be drawn because Z curve crossed both the conventional boundary and the TSA boundary. DEX could reduce the incidence of postoperative vomiting from 16% (control group) to 5.8%. Nevertheless, TSA suggested that > 2977 patients are required before firm conclusions can be drawn on the present or absent intervention effect with a 20 % RRR. Therefore, the result that the DEX could significantly reduce the incidence of vomiting comparing with the control group may also be a false positive. In addition, DEX group had less patients than the control group who administered rescue antiemetic. The effect of antivomiting may result in this superiority. However, TSA indicated that more trials were needed before the firm conclusion. Shivering was the most common postoperative complication during the recovery period after general anesthesia.^33^ A recent systematic review with meta-analysis included 18 studies reporting data on incidence of postoperative shivering.^34^ DEX was associated with a lower incidence of postoperative shivering comparing with the control group. In accordance with this review we found that DEX could reduce the incidence of postoperative shivering from 36% (control group) to 6.7%. Moreover, TSA indicated that firm conclusion can be drawn because Z curve crossed both the conventional boundary and the TSA boundary. The antishivering effect of DEX may depend on some functions. It could suppress the spontaneous firing rate of neurons, decrease the central thermosensitivity,^35^ and then reduce the vasoconstriction and shivering thresholds.^36^

The most common adverse event associated with DEX treatment is bradycardia. Two previous studies indicated that DEX may increase the incidence of bradycardia.^6,10^ However, a recent study showed that DEX may not increase the risk of postoperative bradycardia.^37^ In this meta-analysis, 10 trials including 595 patients showed that DEX group had a significantly lower heart rate than the control group. In addition, TSA indicated that firm conclusion can be drawn because Z curve crossed both the conventional boundary and the TSA boundary. In this meta-analysis, DEX group showed a significantly lower MAP than the control group. TSA indicated that firm conclusion can be drawn because Z curve crossed both the conventional boundary and the TSA boundary. Postoperative hypotension is another common adverse event during DEX treatment, which increases the risk of cerebral ischemia because regulation of cerebral blood perfusion is often impaired near surgically traumatized areas.^38^ Peng et al^37^ found that there was no significant difference in the incidence of treatment for postoperative hypotension between DEX and placebo. Thus, doctors should pay attention to the hypotension when DEX was adopted. No significance was observed between 2 groups about extubation time, which was consistent with previous study.^37^ However, heterogeneity was observed, and sensitivity analyses showed that DEX group had a longer extubation time than control group after omitting “Bakhamees 2007.” The TSA indicated that Z curve crossed the conventional boundary only. Thus, more studies are needed in future before drawing a firm conclusion.

This meta-analysis has some limitations. First, significant heterogeneities and publication bias were observed in some analyses (heart rate, MAP, and extubation time). Second, DEX doses varied from study to study. Outcomes after the highest DEX dose administration were extracted when a trial studied different DEX doses. However, this may affect the results of the meta-analysis.

In conclusion, the present meta-analysis indicated that the administration of DEX may prevent the incidence of postoperative nausea and shivering comparing with the control group. In addition, DEX may cause adverse effects such as lower heart rate, MAP, and longer extubation time. However, more RCTs are needed to make firm conclusion about those adverse effects according to the TSA.

## Supplementary Material

Supplemental Digital Content

## References

[1] Golembiewski J Chernin E Chopra T Prevention and treatment of postoperative nausea and vomiting. *Am J Health Syst Pharm* 2005; 62:1247–1260. 15947124 10.1093/ajhp/62.12.1247

[2] Alfonsi P Postanaesthetic shivering. Epidemiology, pathophysiology and approaches to prevention and management. *Minerva Anestesiol* 2003; 69:438–442. 12768180

[3] Ho CM Wu HL Ho ST Dexamethasone prevents postoperative nausea and vomiting: benefit versus risk. *Acta Anaesthesiol Taiwan* 2011; 49:100–104. 21982171 10.1016/j.aat.2011.06.002

[4] Shakya S Chaturvedi A Sah BP Prophylactic low dose ketamine and ondansetron for prevention of shivering during spinal anaesthesia. *J Anaesthesiol Clin Pharmacol* 2010; 26:465–469. 21547171 PMC3087257

[5] Hwang W Lee J Park J Dexmedetomidine versus remifentanil in postoperative pain control after spinal surgery: a randomized controlled study. *BMC Anesthesiol* 2015; 15:21. 25750586 10.1186/s12871-015-0004-1 PMC4352285

[6] Blaudszun G Lysakowski C Elia N Effect of perioperative systemic alpha2 agonists on postoperative morphine consumption and pain intensity: systematic review and meta-analysis of randomized controlled trials. *Anesthesiology* 2012; 116:1312–1322. 22546966 10.1097/ALN.0b013e31825681cb

[7] Mantz J Josserand J Hamada S Dexmedetomidine: new insights. *Eur J Anaesthesiol* 2011; 28:3–6. 20881501 10.1097/EJA.0b013e32833e266d

[8] Yu C Jia DL Zhang XQ Effects of dexmedetomidine on anxiety in infertile patients after general anesthesia. *Chin J New Drugs* 2014; 23:1665–1668.

[9] Kim YS Kim YI Seo KH Optimal dose of prophylactic dexmedetomidine for preventing postoperative shivering. *Int J Med Sci* 2013; 10:1327–1332. 23983593 10.7150/ijms.6531 PMC3752720

[10] Liu ZX Xu FY Liang X Efficacy of dexmedetomidine on postoperative shivering: a meta-analysis of clinical trials. *Can J Anaesth* 2015; 62:816–829. 25851018 10.1007/s12630-015-0368-1

[11] Watcha MF White PF Postoperative nausea and vomiting. Its etiology, treatment, and prevention. *Anesthesiology* 1992; 77:162–184. 1609990 10.1097/00000542-199207000-00023

[12] Moher D Liberati A Tetzlaff J Preferred reporting items for systematic reviews and meta-analyses: the PRISMA statement. *BMJ* 2009; 339:b2535. 19622551 10.1136/bmj.b2535 PMC2714657

[13] Higgins JP Green S Cochrane Handbook for Systematic Reviews of Interventions 5.1.0 [Updated March 2011]. The Cochrane Collaboration. Available at: http://www.cochrane.org/training/cochrane-handbook.

[14] Jakobsen JC Wetterslev J Winkel P Thresholds for statistical and clinical significance in systematic reviews with meta-analytic methods. *BMC Med Res Methodol* 2014; 14:120. 25416419 10.1186/1471-2288-14-120 PMC4251848

[15] Guyatt GH Oxman AD Vist GE GRADE: an emerging consensus on rating quality of evidence and strength of recommendations. *BMJ* 2008; 336:924–926. 18436948 10.1136/bmj.39489.470347.AD PMC2335261

[16] Higgins JP Thompson SG Deeks JJ Measuring inconsistency in meta-analyses. *BMJ* 2003; 327:557–560. 12958120 10.1136/bmj.327.7414.557 PMC192859

[17] DerSimonian R Laird N Meta-analysis in clinical trials. *Control Clin Trials* 1986; 7:177–188. 3802833 10.1016/0197-2456(86)90046-2

[18] Turner RM Bird SM Higgins JP The impact of study size on meta-analyses: examination of underpowered studies in Cochrane reviews. *PloS One* 2013; 8:e59202. 23544056 10.1371/journal.pone.0059202 PMC3609745

[19] Aho M Scheinin M Lehtinen AM Intramuscularly administered dexmedetomidine attenuates hemodynamic and stress hormone responses to gynecologic laparoscopy. *Anesth Analg* 1992; 75:932–939. 1359808

[20] Bakhamees HS El-Halafawy YM El-Kerdawy HM Effects of dexmedetomidine in morbidly obese patients undergoing laparoscopic gastric bypass. *Middle East J Anaesthesiol* 2007; 19:537–551. 18044282

[21] Tufanogullari B White PF Peixoto MP Dexmedetomidine infusion during laparoscopic bariatric surgery: the effect on recovery outcome variables. *Anesth Analg* 2008; 106:1741–1748. 18499604 10.1213/ane.0b013e318172c47c

[22] Massad IM Mohsen WA Basha AS A balanced anesthesia with dexmedetomidine decreases postoperative nausea and vomiting after laparoscopic surgery. *Saudi Med J* 2009; 30:1537–1541. 19936416

[23] Bhattacharjee DP Nayek SK Dawn S Effects of dexmedetomidine on haemodynamics in patients undergoing laparoscopic cholecystectomy—a comparative study. *J Anaesthesiol Clin Pharmacol* 2010; 26:45–48. PMC3087277 21547174

[24] Singh Bajwa SJ Gupta S Kaur J Reduction in the incidence of shivering with perioperative dexmedetomidine: a randomized prospective study. *J Anaesthesiol Clin Pharmacol* 2012; 28:86–91. 22345953 10.4103/0970-9185.92452 PMC3275980

[25] Karaman S Gunusen I Ceylan MA Dexmedetomidine infusion prevents postoperative shivering in patients undergoing gynecologic laparoscopic surgery. *Turkish J Med Sci* 2013; 43:232–237.

[26] Wu Y Huang H Zeng J Effect of dexmedetomidine in preventing shivering after general anesthesia for laparoscopic surgery: a randomized, single-blinded, and placebo-controlled trial. *Nan Fang Yi Ke Da Xue Xue Bao* 2013; 33:611–614. 23644132

[27] Bhanderi D Shah C Shah B Comparison of iv Dexmedetomidine V/S iv Clonidine in hemodynamic stability in laparoscopic surgery. *Res J Pharm Biol Chem Sci* 2014; 5:910–917.

[28] Manne GR Upadhyay MR Swadi VN Effects of low dose dexmedetomidine infusion on haemodynamic stress response, Sedation and post-operative analgesia requirement in patients undergoing laparoscopic cholecystectomy. *Indian J Anaesth* 2014; 58:726–731. 25624537 10.4103/0019-5049.147164 PMC4296358

[29] Seo KH Kim YI Kim YS Optimal dose of dexmedetomidine for attenuating cardiovascular response during emergence in patients undergoing total laparoscopic hysterectomy. *J Int Med Res* 2014; 42:1139–1149. 25004921 10.1177/0300060514531925

[30] Kim NY Yoo YC Park H The effect of dexmedetomidine on intraocular pressure increase in patients during robot-Assisted laparoscopic radical prostatectomy in the steep trendelenburg position. *J Endourol* 2015; 29:310–316. 25137430 10.1089/end.2014.0381

[31] Srivastava VK Nagle V Agrawal S Comparative evaluation of dexmedetomidine and esmolol on hemodynamic responses during laparoscopic cholecystectomy. *J Clin Diagn Res* 2015; 9:UC01–UC5. 25954683 10.7860/JCDR/2015/11607.5674 PMC4413133

[32] Bakri MH Ismail EA Ibrahim A Comparison of dexmedetomidine and dexamethasone for prevention of postoperative nausea and vomiting after laparoscopic cholecystectomy. *Korean J Anesthesiol* 2015; 68:254–260. 26045928 10.4097/kjae.2015.68.3.254 PMC4452669

[33] Shen H Chen Y Lu KZ Parecoxib for the prevention of shivering after general anesthesia. *J Surg Res* 2015; 197:139–144. 25908099 10.1016/j.jss.2015.03.011

[34] Piao G Wu J Systematic assessment of dexmedetomidine as an anesthetic agent: a meta-analysis of randomized controlled trials. *Arch Med Sci* 2014; 10:19–24. 24701209 10.5114/aoms.2014.40730 PMC3953974

[35] Bajwa SJ Gupta S Kaur J Reduction in the incidence of shivering with perioperative dexmedetomidine: a randomized prospective study. *J Anaesthesiol Clin Pharmacol* 2012; 28:86–91. 22345953 10.4103/0970-9185.92452 PMC3275980

[36] Talke P Tayefeh F Sessler DI Dexmedetomidine does not alter the sweating threshold, but comparably and linearly decreases the vasoconstriction and shivering thresholds. *Anesthesiology* 1997; 87:835–841. 9357885 10.1097/00000542-199710000-00017

[37] Peng K Wu S Liu H Dexmedetomidine as an anesthetic adjuvant for intracranial procedures: meta-analysis of randomized controlled trials. *J Clin Neurosci* 2014; 21:1951–1958. 24974190 10.1016/j.jocn.2014.02.023

[38] Fieschi C Agnoli A Battistini N Derangement of regional cerebral blood flow and of its regulatory mechanisms in acute cerebrovascular lesions. *Neurology* 1968; 18:1166–1179. 4303018 10.1212/wnl.18.12.1166

