# Supplementary material for: Effect of Dexmedetomidine in Preventing Postoperative Side Effects for Laparoscopic Surgery: A Meta-Analysis of Randomized Controlled Trials and Trial Sequential Analysis (PRISMA)
Source: Medicine (Baltimore). 2016 Mar 11;95(10):e2927. doi: 10.1097/MD.0000000000002927 (PMC4998870; doi:10.1097/MD.0000000000002927)

Electronic supplementary material (ESM) content **Page**

ESM 1: Figure 13 Trial sequential analysis of rescue antiemetic……….. 1

Figure 13 Trial sequential analysis of rescue antiemetic

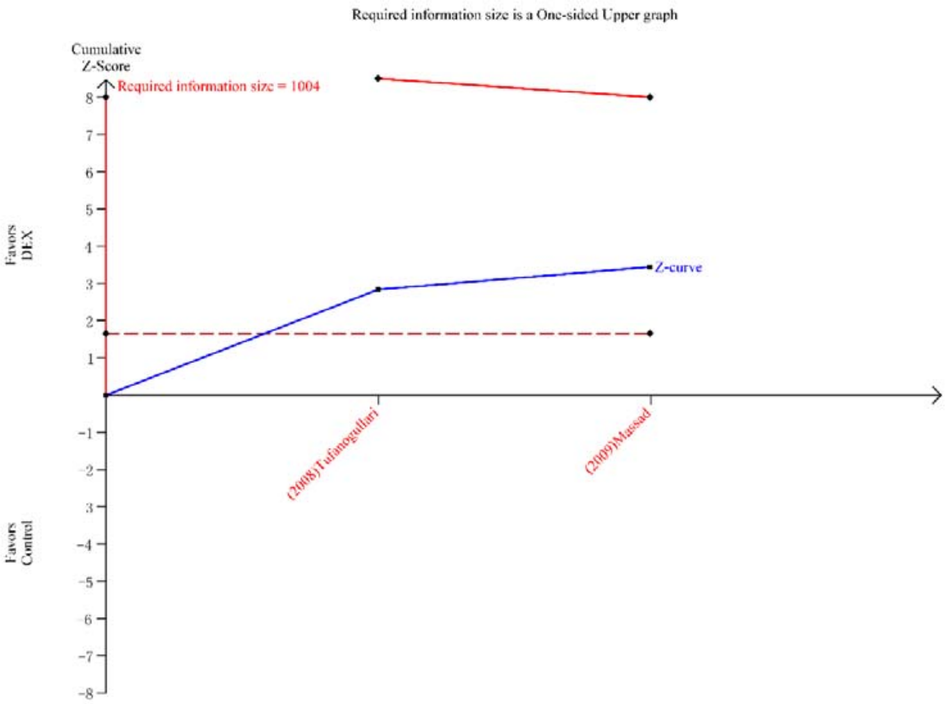

Electronic supplementary material (ESM) content **Page**

1. ESM 2: Figure 14 Trial sequential analysis of dry mouth……………..…1

Figure 14 Trial sequential analysis of dry mouth

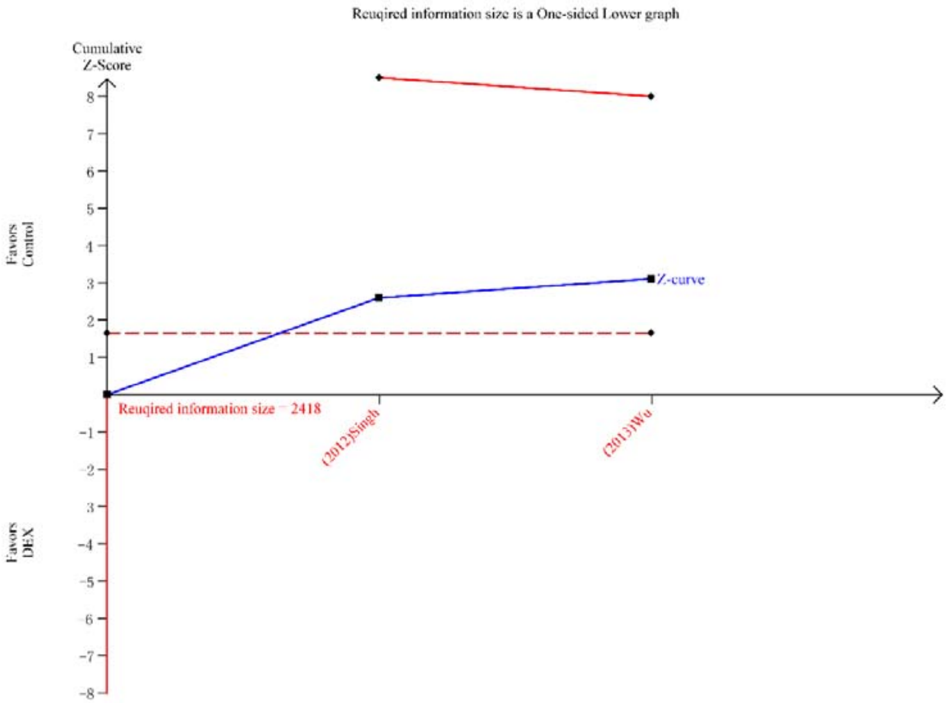

Electronic supplementary material (ESM) content **Page**

1. ESM 3: Figure 15 Trial sequential analysis of extubation time………….. 1

Figure 15 Trial sequential analysis of extubation time

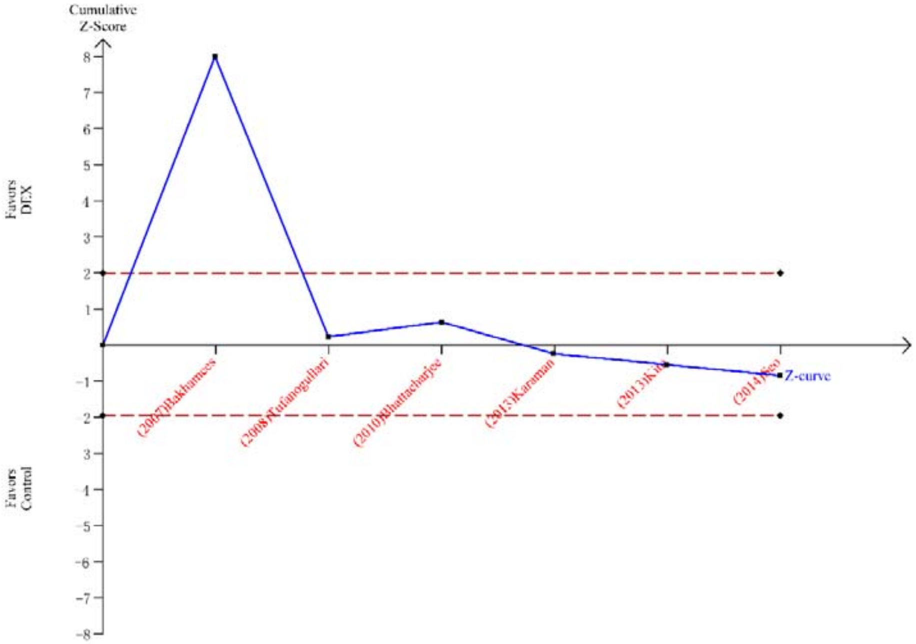

Electronic supplementary material (ESM) content **Page**

1. ESM 4: Figure 16 Publication bias of heart rate………………………….1

Figure 16 Publication bias of heart rate

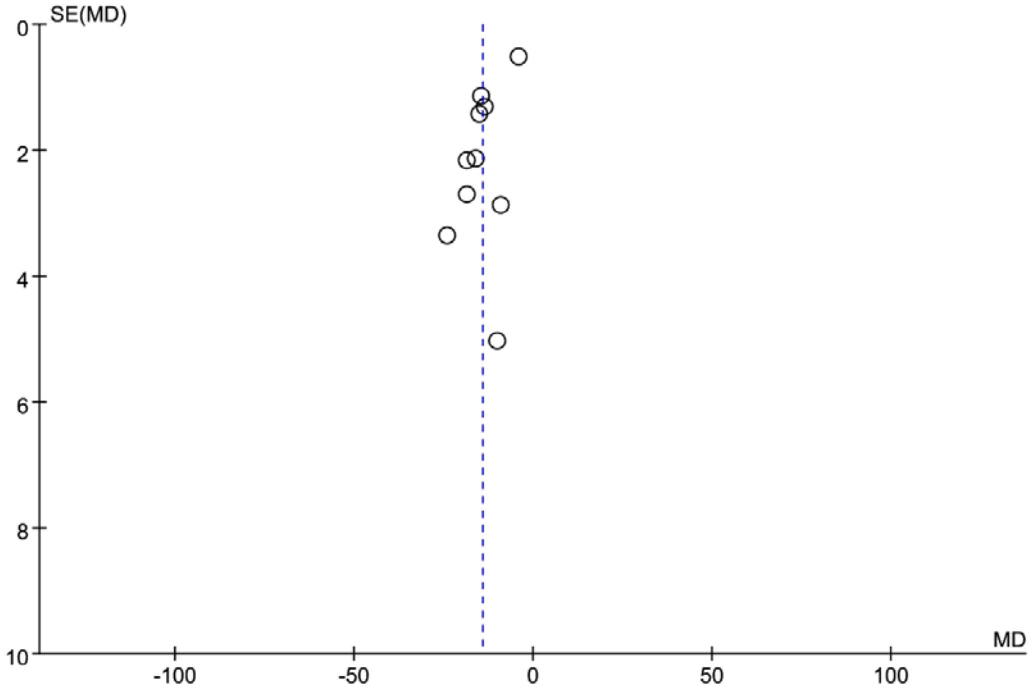

Electronic supplementary material (ESM) content **Page**

1. ESM 5: Figure 17 Publication bias of MAP……………………….…….1

Figure 17 Publication bias of MAP

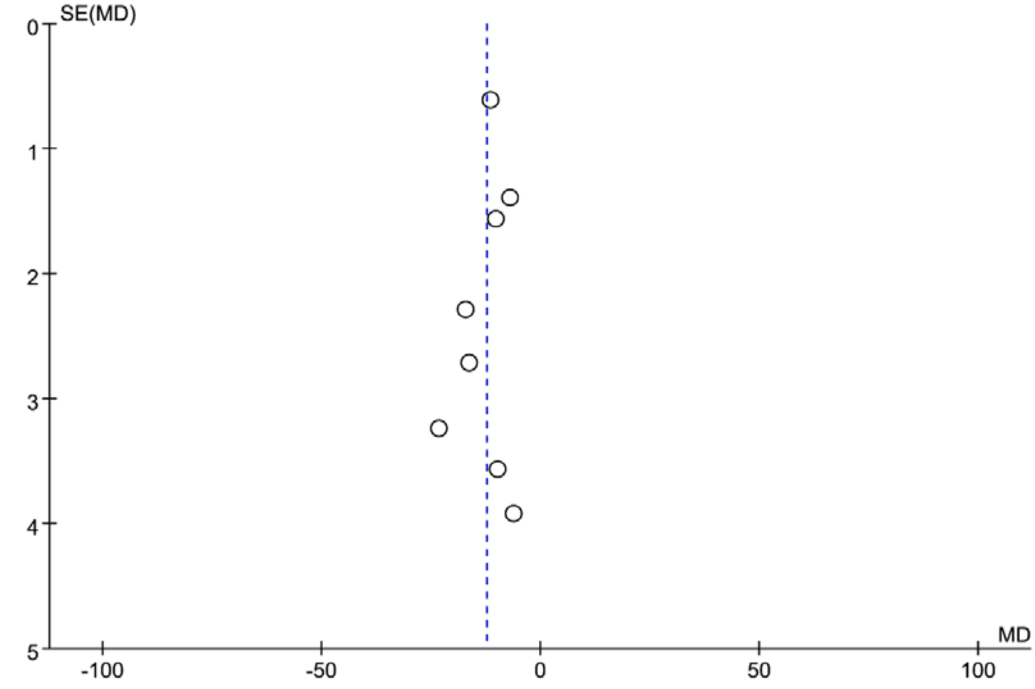

Electronic supplementary material (ESM) content **Page**

1. ESM 6: Figure 18 Publication bias of Extubation time………………..…… 1

Figure 18 Publication bias of Extubation time

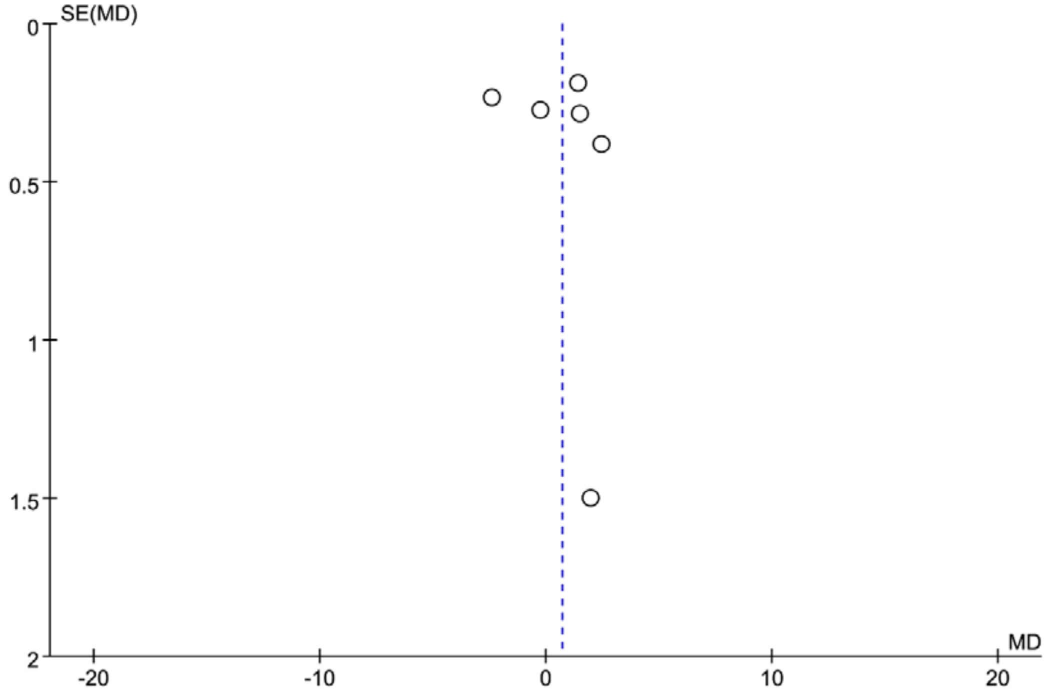

Electronic supplementary material (ESM) content Page

1. ESM 7: Figure 19 Sensitivity analyses of Heart rate…………….………… 1

Figure 19 Sensitivity analysis of Heart rate

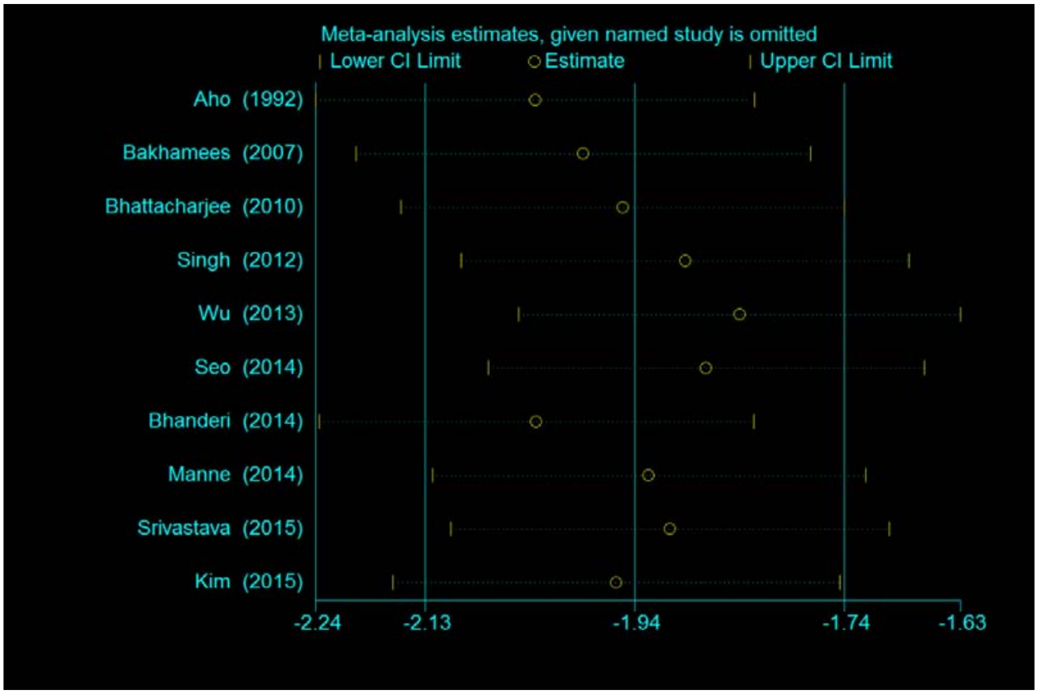

Electronic supplementary material (ESM) content **Page**

1. ESM 8: Figure 20 Sensitivity analyses of MAP………………………… 1

Figure 20 Sensitivity analysis of MAP

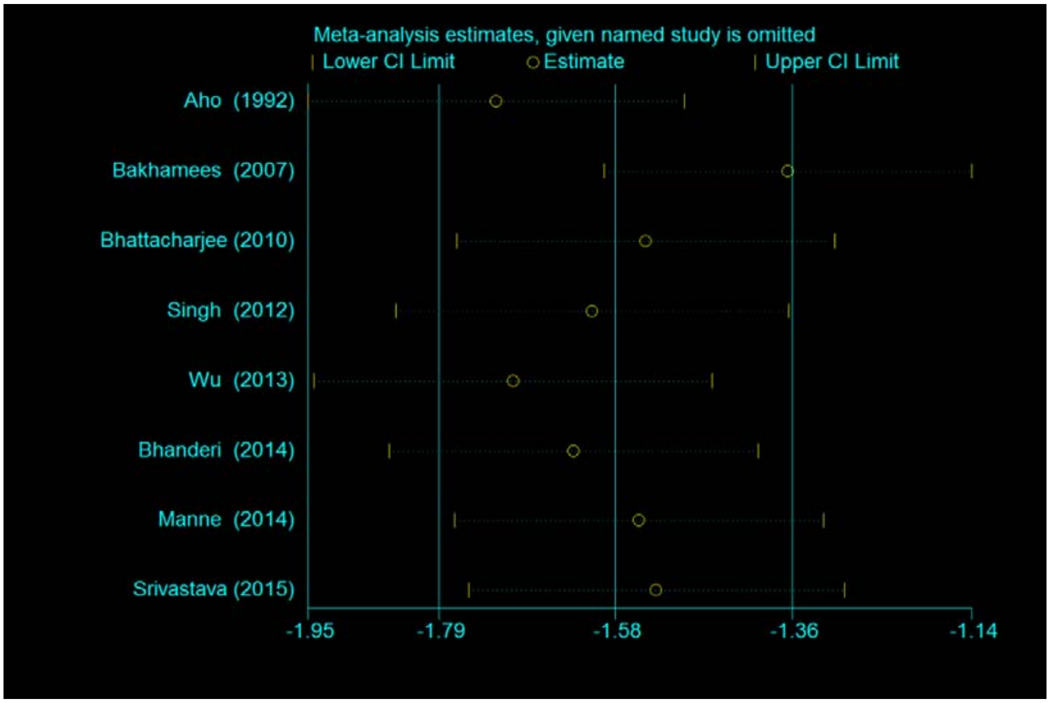

Electronic supplementary material (ESM) content **Page**

1. ESM 9: Figure 21 Sensitivity analyses of Extubation time…………..….1

Figure 21 Sensitivity analysis of Extubation time

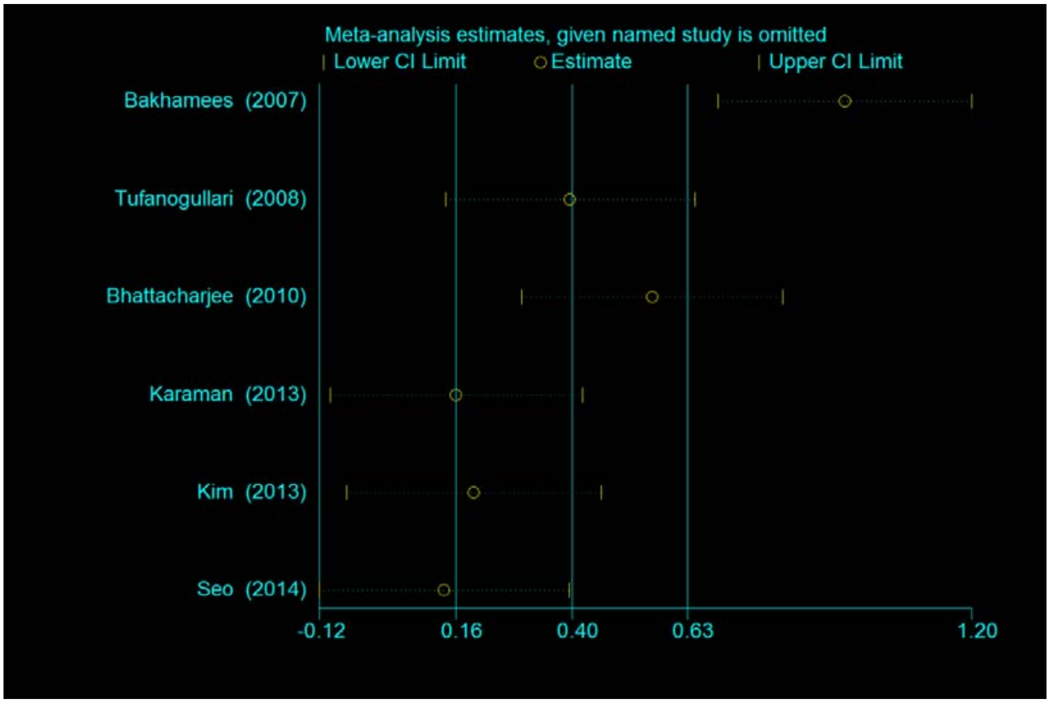

Supplement: Supplemental Digital Content [file medi-95-e02927-s001.pdf]
